# Characterization of Metabolites and Transcriptome of Pepper Accessions from Four Southern Provinces of China

**DOI:** 10.3390/genes16020137

**Published:** 2025-01-24

**Authors:** Zhuo Liu, Chuangchuang Yang, Jianwen He, Lingkui Zhang, Xiaolin Xing, Kang Zhang, Hailong Yu, Zhenghai Zhang, Huamao Wu, Feng Cheng, Yacong Cao, Lihao Wang

**Affiliations:** 1 State Key Laboratory of Vegetable Biobreeding, Institute of Vegetables and Flowers, Chinese Academy of Agricultural Sciences, Beijing 100081, China; 17700572140@163.com (Z.L.); yangcc503@163.com (C.Y.); zhanglk960127@163.com (L.Z.); 821012410270@caas.cn (X.X.); zhangkang01@caas.cn (K.Z.); yuhailong@caas.cn (H.Y.); zhangzhenghai@caas.cn (Z.Z.); wuhuamao@caas.cn (H.W.); chengfeng@caas.cn (F.C.); wanglihao@caas.cn (L.W.); 2 College of Plant Science and Technology, Beijing University of Agriculture, Beijing 100096, China; 3 Guizhou Pepper Research Institute, Guiyang 550006, China; hejianwen1022@126.com

**Keywords:** pepper, metabolite profiling, transcriptome, flavonoid biosynthesis, regional variation

## Abstract

Background/Objectives: Pepper (*Capsicum annuum* L.) is a widely grown vegetable and spice crop worldwide. This study aims to reveal the differences of metabolites among pepper accessions from different regions and explore candidate genes related to metabolites of pepper. Methods: The metabolome and transcriptome of 36 pepper accessions were determined by widely targeted metabolomics analysis and RNA sequencing technology, and the differential metabolites and differential genes among *C. annuum* from four important pepper production and consumption provinces of China, Hunan, Guizhou, Yunnan and Sichuan, were analyzed. Results: Flavonoids are the main characteristic metabolites that distinguish pepper accessions from Yun_Gui_Chuan Group and Hunan Group. The aglycones of characteristic flavonoids in each group are different; in Yun_Gui_Chuan Group mainly are luteolin, quercetin, chrysoeriol and isorhamnetin; in Hunan Group mainly are apigenin. Transcriptome data showed that two genes related to flavonoid 3′-monooxygenase differed significantly between the two groups of chili peppers, and we speculated that they may be the core enzymes regulating their flavonoid profile. And an SNP mutation located in gene *Cgla06g001871* showed a strong correlation with pepper accessions from Yun_Gui_Chuan Group, which can be used as a DNA marker to identify pepper accessions from Yun_Gui_Chuan Group, and provide strong support for regional specialty variety conservation. In addition, we also analyzed the metabolites related to the taste and nutrition of pepper accessions in the four provinces, and the results showed that the sugar content of pepper accessions from Guizhou was low and the capsaicinoids content of pepper accessions from Sichuan was low, while no significant difference was found in acid and vitamin contents among pepper accessions from the four provinces. Conclusions: The metabolome and transcriptome of 36 pepper accessions from four important pepper production and consumption provinces of China were determined, and the characteristic metabolites and expressed genes of pepper accessions from each province were analyzed.

## 1. Introduction

Pepper (*Capsicum spp.*), which primarily originated in the Central and South American regions [1,2], belongs to the genus *Capsicum* within the *Solanaceae* Juss. family. It is cultivated and consumed globally as both a fresh vegetable and flavoring spice. Pepper is not only an important vegetable but also a major spice crop globally, possessing crucial economic and social value along with its culinary value that has been recognized for centuries [3]. According to the report of the Food and Agriculture Organization of the United Nations (FAO) (https://www.fao.org), the global area granted to chili pepper cultivation in 2022 is 3,666,300 hectares, yielding a total production of 41,881,800 metric tons and an overall output value of 46,281 million U.S. dollars, highlighting its considerable economic and social importance. In the 21st century, the global planting area and production of peppers have increased consistently, and China’s pepper industry has also entered a phase of rapid growth.

Pepper is intensely cherished by people due to its nutritious quality and spicy flavor, and the number of individuals who appreciate eating pepper continues to grow, resulting in a steady increase in the scale of pepper cultivation. It has been cultivated for more than 6000 years. It is traditionally believed that Columbus brought chili peppers from South America to Europe in 1493 and then to Asia, Africa and Oceania [4,5]. Pepper was widely distributed around the world during the sixteenth and seventeenth centuries [6], and it was introduced to China during Ming Period [7], where it was rapidly distributed throughout the nation.

As a popular vegetable and seasoning crop, pepper is numerous sources of nutrients including vitamins and flavonoids [8] with a wide range of taste flavors ranging from sweet to spicy [9], also displaying vigorous colors including green, yellow, red, white and purple [10]. Flavonoids are important secondary metabolites found in pepper fruits, generally with C6-C3-C6 as the basic carbon framework, capable of combining with sugars in the organism to form glycosides or carbonyl glycosides, and only a few exist in the free form. They can be divided into the following categories: anthocyanins, flavonoids, flavanones, flavanols, isoflavones, chalcones, dihydrochalcones and flavonoid glycosides [11], and they possess anticancer, anti-inflammatory, antioxidant and immune-enhancing properties [12,13,14,15]. This has led to an increasing focus on research concerning the quality and breeding of chili peppers [16]. Sugar and organic acids play crucial roles as metabolites in pepper fruits, and they are linked to factors like material composition and the stage of fruit ripening [17]. In comparison to tomatoes and eggplants, the pepper has the highest average level of total sugars and organic acids, with the primary sugars identified as fructose and glucose and citric acid being the predominant organic acids [18]. Capsaicinoids are secondary metabolites particularly found in pepper fruits, responsible for their distinctive spiciness and possessing numerous established health benefits and industrial uses [19]. L-ascorbic acid (vitamin C), serving various functions including nutrition, medical treatment and health care, is crucial for sustaining normal physiological functions and health in human, contributing to improving immune function, anti-oxidation, anti-aging and cancer prevention [20,21].

Regarding the differences of plant metabolites in different regions, there were some studies on crops such as quinoa [22] and rice [23]. But fewer studies on peppers and the differences in metabolites of different phenotypes [24] and varieties [25] were mainly studied in peppers. Plant domestication is a complex and lengthy process that involves human selection, cultivation and improvement of wild plants to meet specific needs [26]. In this process, environmental factors play a crucial role. Different geographical environments and climatic conditions have profound effects on the growth, reproduction and adaptability of plants [27,28,29,30]. Environmental factors such as temperature, light, moisture, soil type and biodiversity have an impact on plant growth and development [31,32,33,34]. These environmental factors play a role in screening and shaping the process of plant domestication. For example, in arid regions, plants may adapt to their environment by evolving deeper root systems or more drought-tolerant physiological mechanisms [35].

Hunan [36], Yunnan [37], Guizhou [38] and Sichuan [39] are leading provinces in China regarding production and consumption of peppers. Previous researchers have analyzed the development of different tissues [40], the differences in carotenoid [41] and capsianoside metabolites [42] through metabolome analysis and transcriptome analysis in peppers. Metabolome and transcriptome analyses were conducted on pepper varieties from these four provinces to investigate the unique metabolites and expressed genes of pepper accessions originating from various regions, providing a foundation for the identification of different pepper accessions.

## 2. Materials and Methods

### 2.1. Materials

Thirty-six pepper accessions (Table S1) including 10 accessions from Hunan, 8 accessions from Guizhou, 6 accessions from Sichuan and 12 accessions from Yunnan provinces were planted in a greenhouse, located in Beijing at 40°10′ N latitude and 116°52′ E longitude in the spring of 2021, and the plants were cultivated and managed uniformly. Fruit samples were collected at the break stage and mature stage, respectively. At each stage, 3–10 fruits were collected each accession, depending on the fruit size. The stalks and seeds were removed and stored in a −80 °C refrigerator after being rapidly frozen in liquid nitrogen for at least 30 s until determination.

### 2.2. Widely Targeted Metabolomics Analysis

Two replicates were made for each accession. Fruit samples of peppers at the mature stage were taken out from an ultra-low temperature (−80 °C) refrigerator, freeze-dried, ground into powder, accurately weighed to 0.1 g of powder, added to 1 mL of 70% aqueous methanol (containing 1 ug/mL 2-chlorophenylalanine as the internal standard), vortexed for 1 min and extracted at 4 °C for 303 min, with vortexing three times during the extraction process. After that, it was centrifuged at 12,000 r/min at 4 °C for 10 min. The supernatant was transferred to an injection vial for liquid chromatography–mass spectrometry/mass spectrometry (LC–MS/MS) analysis. The main instruments used for the tests were as follows: an ultra-high performance liquid chromatograph model Shim pack UFLC SHIMADZU CBM30A from Shimadzu, a tandem mass spectrometer (MS/MS) and a QTRAP ^®^ 4500+ (https://sciex.com/). The analytical conditions of liquid chromatography were as follows: (1) chromatographic column (Waters ACQUITY UPLC HSS T3 C18 1.8 µm, 92.1 mm × 100 mm); (2) ultra-pure water (containing 0.1% formic acid) for phase A and acetonitrile (containing 0.1% formic acid) for phase B; (3) elution gradient: the eluent was a mixture of water and acetonitrile, and the volume ratio of water and acetonitrile was 95:5 at 0 min, 5:95 at 10 min and 5:95 at 11 min and 11.1 min. The volume ratio of water and acetonitrile was 95:5 at 0 min, water and acetonitrile at 10 min, water and acetonitrile at 11 min, water and acetonitrile at 11.1 min and water and acetonitrile at 15.0 min. (4) The flow rate was set at 0.4 mL per minute, and the column temperature was set at 40 degrees Celsius, and the injection volume of each sample was 2 microliters. The key parameters for the mass spectrometry analysis were set as follows: the temperature of the electrospray ionization source was maintained at 550 degrees Celsius; the positive and negative mass spectrometry voltages were set to 5500 volts and 4500 volts, respectively; and the pressure of the ionization source gas I was controlled to be 55 psi, that of the ionization source gas II was set to be 60 psi, and the pressure of the curtain gas was set to be 25 psi. Also, the collision activated ionization parameter was set to a high level. In a triple quadrupole (Qtrap) instrument, each ion pair was carefully scanned and detected by precisely modulating the collision energy and optimizing the clustering potential [43]. The metabolites were accurately analyzed qualitatively based on the retention time of the detected substances, the information of the parent ion pairs and the secondary spectral data compared with the self-constructed target standard database. Quantitative analysis of metabolites was performed by integrating the ion chromatography peaks under the peak area and correcting the integrals to eliminate possible bias between samples [44].

In MetaboAnalyst 6.0 (https://www.metaboanalyst.ca), the metabolomic data were subjected to sum normalization, logarithmic transformation (with base 10) and auto scaling to make the metabolomics data approximately conform to a normal distribution, and the obtained data were statistically analyzed [45]. Differences in metabolites of pepper accessions from the four provinces were investigated using unsupervised principal component analysis (PCA). The Fold Change (FC) and false discovery rate (FDR) of metabolites were obtained from volcano plot analysis, and the variable importance in the projection (VIP) values obtained by supervised orthogonal partial least squares discriminant analysis (oPLS-DA) were used to mine differential metabolites. Supervised partial least squares discriminant analysis (PLS-DA) was used to describe the differences in the metabolic profiles of the three source provinces in Yunnan, Guizhou and Sichuan. The screening criteria [46] for metabolites with differential expression were as follows: (1) FC > 1.5 or FC < 0.75; (2) FDR *p* < 0.05; and (3) VIP > 1.5.

### 2.3. RNA-Seq and Resequencing

Pepper fruit samples at the break stage were used for high-throughput transcriptome sequencing at Beijing Genomics Institute (BGI, Shenzhen, China), and the sequencing and analysis process was performed according to the standard protocols of BGI RNA-seq [47,48,49]. The transcriptome sequencing data ranged from 7.1 Gb to 36.8 Gb with a mean value of 17.3 Gb. Clean reads were estimated for the relative expression of transcripts per kilobase of exon model per million mapped reads (TPM). The mRNA-seq data were mapped to the PM1533 Anu7 genome (not published) using Hisat2 software (version 2.1.0) and Samtools software (version 1.9) using default parameters, and the identified genes were annotated through the Arabidopsis Genome Resource Database (https://www.arabidopsis.org). The selection criteria for differential genes are the same as for differential metabolites: (1) FC > 1.5 or FC < 0.75; (2) FDR *p*< 0.05; and (3) VIP > 1.5.

The genome resequencing data are the data from our previous published article [50]. Thirty-six accessions resequencing data studied in this research were used for drawing an NJ tree by phylip software and then beautified in ITOL (https://itol.embl.de/).

### 2.4. Stastatistical Analysis

The statistical analyses were performed by the SPSS 26.0 statistical package (IBM, Armonk, NY, USA). Continuous variables that followed a normal distribution are presented as the mean ± standard deviation, while those that did not follow a normal distribution are presented as the median and inter-quartile range; categorical variables are represented as number (%). For comparing multiple groups, *p* values were calculated using one-way ANOVA for continuous variables or the chi-square test for categorical variables. A *p* value of less than 0.05 was statistically significant for all comparisons. The R ggplot2 package in R 4.4.1 was used to create the boxplot [51].

## 3. Results

### 3.1. Genome, Transcriptome and Metabolome Analyses

**Figure 1:**
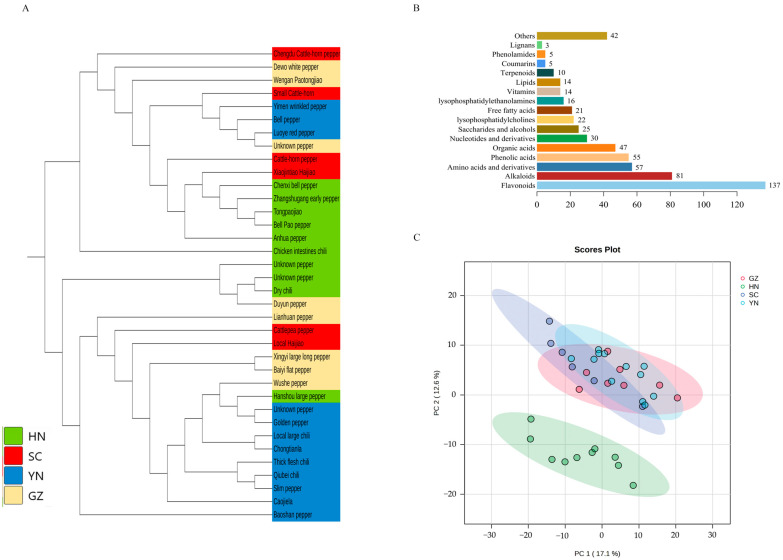
Genome and metabolome analyses. (**A**): The neighbor joining tree of 36 pepper accessions. (**B**): The classification of 584 metabolites. (**C**): Principal component analysis of pepper metabolites from four provinces (HN-Hunan; SC-Sichuan; YN-Yunnan; GZ-Guizhou).

The resequencing data were obtained from our previous study [50], and the neighbor joining tree was constructed with 36 accessions (Figure 1A). Although the domestication of *C. annuum* was chaotic, the results showed that pepper accessions from different provinces were roughly clustered together, especially the accessions from Hunan and Yunnan.

The transcriptome data were aligned with the reference genome PM1533 Anu7 at rates ranging from 57.52% to 72.92%, with an average alignment rate of 67.96%, and a total of 36,010 expressed genes were identified in 36 accessions.

A total of 622 metabolites were detected, and 584 metabolites (Table S2) were obtained by excluding metabolites with coefficients of variation (CV) greater than 30% in the quality control (QC) samples by referring to the method of Tang [52]. The classification of 584 metabolites was visualized, which contained 137 flavonoids, 81 alkaloids, 57 amino acids and derivatives, 55 phenolic acids, 47 organic acids, 30 nucleotides and derivatives, 25 saccharides and alcohols, 22 Lys phosphatidylcholines, 21 free fatty acids, 16 Lys phosphatidylethanolamines, 14 vitamins, 14 lipids, 10 terpenoids, five coumarins, five phenol amides, three lignans and 42 other metabolites (Figure 1B, Table S2).

### 3.2. Characteristic Differential Metabolites and Expressed Genes of Pepper Accessions from Hunan Group and Yun_Gui_Chuan Group

According to PCA, oPLS-DA and FC of metabolites content analyses, flavonoids were the main metabolites that distinguish pepper accessions from Hunan province and Yunnan, Guizhou and Sichuan provinces.

**Figure 2:**
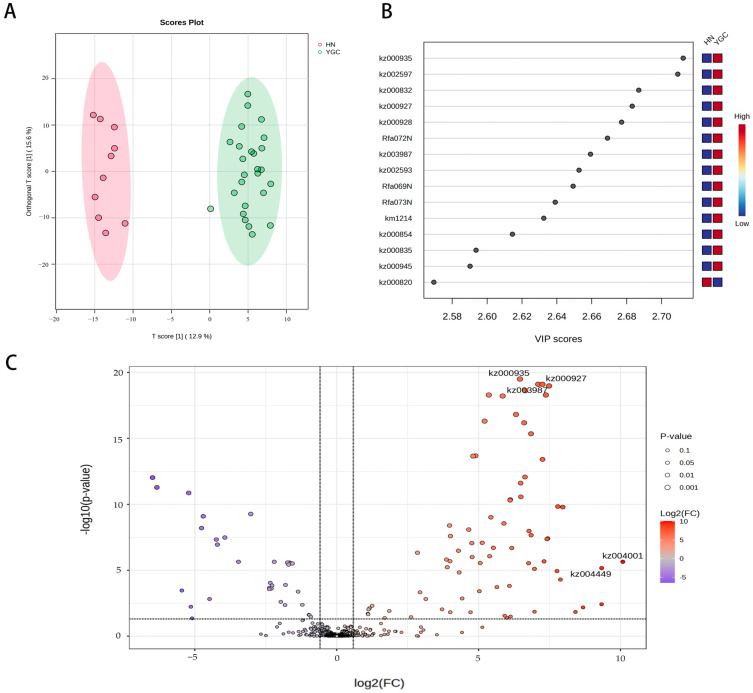
Metabolites analyses of pepper accessions from Hunan and Yun_Gui_Chuan Groups. (**A**): oPLS-DA score chart; (**B**): oPLS-DA VIP score chart; (**C**): volcano plot.

The PCA analysis based on the standardized data of metabolites showed that under the condition of no grouping supervision, 36 accessions were clearly divided into two clusters on PC2 (Figure 1C), the cluster whose accessions are from Hunan province was named Hunan Group, and the other cluster whose accessions are from Yunnan, Guizhou and Sichuan provinces was named Yun_Gui_Chuan Group. The main contributing annotated metabolites were kaempferol-C-glucoside-C-glucoside, C-hexosyl-luteolin C-pentoside, luteolin-6,8-di-C-glucoside, Chrysoeriol 6-C-glucoside (isoscoparin), chrysoeriol 8-C-hexoside, luteolin-6-C-glucoside (isoorientin), luteolin 6-C-hexoside-8-C-pentoside, luteolin-6-C-2-glucuronylglucoside, 6-C-hexosyl chrysoeriol O-hexoside, apigenin O-hexosyl-O-pentoside and luteolin-O-Malonyl-O-hexoside-O-pentoside in PC 2. Orthogonal partial least squares discriminant analysis (oPLS-DA) of metabolites of Hunan Group and Yun_Gui_Chuan Group showed that the two groups were separated on component 1 (Figure 2A), and R2 and Q2 were greater than 0.85 due to cross validation. There were 89 metabolites with the VIP value greater than 1.5 in component 1, and the top 15 metabolites with the highest VIP values are shown in Figure 2B and Table S3, of which 14 flavonoids were higher in pepper accessions from Yun_Gui_Chuan Group and one metabolite, apigenin O-hexosyl-O-pentoside, was higher in pepper accessions from Hunan Group.

According to FC > 1.5 or FC < 0.75, FDR adjusted *p* < 0.05, 75 metabolites contents were higher in pepper accessions from Yun_Gui_Chuan Group and 33 metabolites contents were higher in pepper accessions from Hunan Group (Figure 2C; Table S4). The 75 differential metabolites with higher content in Yun_Gui_Chuan Group were mainly flavonoids, including two anthocyanins, 17 flavanones, 35 flavonols, 12 flavonoid carbonosides, one flavanol and one isoflavone, and also one alkaloid, one amino acid derivative, two Lys phosphatidylcholine (LPC) and two Lys phosphatidylethanolamine (LPE). The 33 metabolites, which were more in pepper accessions from Hunan Group, were also mainly flavonoids, including eight flavanones, 10 flavonols, nine flavonoid carbocosides and two isoflavones, and also four phenolic acids.

Kaempferol-C-glucoside-C-glucoside, C-hexosyl-luteolin C-pentoside, luteolin-6,8-di-C-glucoside, isoscoparin, chrysoeriol 8-C-hexoside, luteolin-6-C-glucoside (isoorientin), luteolin 6-C-hexoside-8-C-pentoside, luteolin-6-C-2-glucuronyl glucoside, luteolin-O-Malonyl-O-hexoside-O-pentoside and apigenin O-hexosyl-O-pentoside were the common differential metabolites according to the analyses of PCA, oPLS-DA and FC, and they are all flavonoids (Table 1). The content of apigenin O-hexosyl-O-pentoside, which is among the common differential metabolites, is higher in accessions from Hunan Group, so apigenin O-hexosyl-O-pentoside is considered the characteristic metabolite in pepper accessions from Hunan Group. And the content of the rest 14 of the common differential metabolites are higher in pepper accessions from Yun_Gui_Chuan Group, so these 14 flavonoids are considered the characteristic metabolites in Yun_Gui_Chuan Group.

Although the differential metabolites between accessions from Hunan Group and Yun_Gui_Chuan Group are mostly flavonoids, the aglycone of the differential flavonoids are different. The flavonoids with more content in pepper accessions from Yun_Gui_Chuan Group are mainly luteolin, quercetin, chrysoeriol and isorhamnetin; while flavonoid with more contents in pepper accessions from Hunan Group is apigenin. In the flavonoid biosynthesis processes, the luteolin and chrysoeriol, which were more abundant in Yun_Gui_Chuan Group, are located in the downstream of apigenin, which is more abundant in Hunan Group.

**Figure 3:**
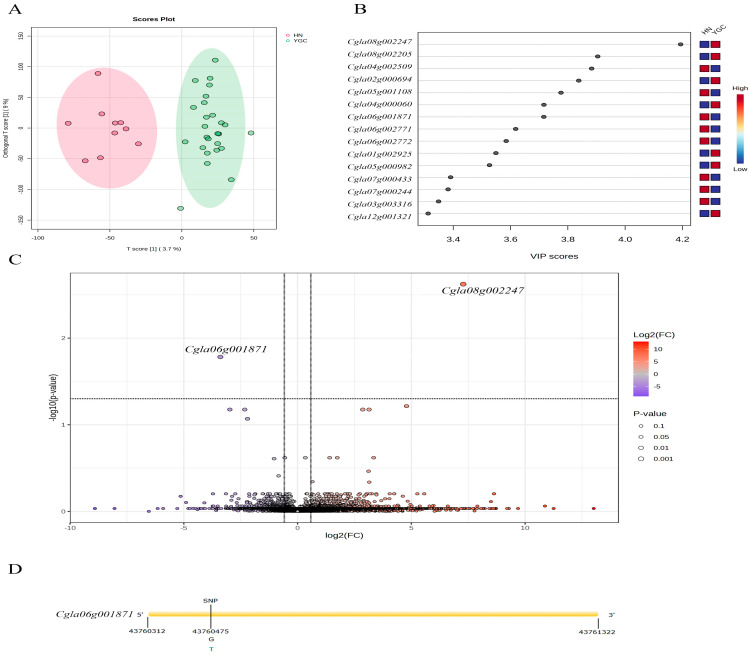
Hunan (HN) Group and Yun_Gui_Chuan(YGC) Group transcriptome analyses. (**A**): oPLS-DA score chart; (**B**): oPLS-DA VIP score chart; (**C**) volcano plot; (**D**): the structure of Cgla06g001871 and the SNP position.

Orthogonal partial least squares discriminant analysis (oPLS-DA) of the transcriptomic data of pepper accessions from Hunan Group and Yun_Gui_Chuan Group showed that in the horizontal axis, Hunan Group pepper accessions were distinguished from Yun_Gui_Chuan Group pepper accessions (Figure 3A). The fifteen differential expressed genes with the highest VIP values were shown in Figure 3B and Table 2, and the expression of *Cgla08g002247*, *Cgla08g002205*, *Cgla02g000694*, *Cgla04g000060*, *Cgla01g002925*, *Cgla05g000982* and *Cgla12g001321* was higher in the pepper accessions from Yun_Gui_Chuan Group, and the expression of *Cgla04g002509*, *Cgla05g001108, Cgla06g001871*, *Cgla06g002771*, *Cgla06g002772*, *Cgla07g000433, Cgla07g000244* and *Cgla03g003316* was higher in the pepper accessions from Hunan Group. According to criteria of FC > 1.5 or FC < 0.75, and FDR adjusted *p* <0.05, a significant downregulated gene Cgla06g001871 and an upregulated gene *Cgla08g002247* were obtained (Figure 3C), which were also with the higher VIP value in the OPLS-DA analysis. We annotated the function of pepper genes by homologous alignment with Arabidopsis thaliana genes (Table 2). *Cgla06g001871* encodes the Phorbol-ester/DAG-type domain-containing protein, which is related to signal transduction. An SNP was identified at the position 43760475 bp on chromosome 6 in the gene *Cgla06g001871*, and the base change from G to T, resulting in the amino acid changes from arginine to leucine. In the 36 accessions, 24 accessions had this mutation within gene *Cgla06g001871*, and among the 24 accessions, only one is from Hunan province, and the rest are from Yunnan, Guizhou and Sichuan provinces. So, the SNP at the position 43760475 bp on chromosome 6 can serve as a marker to distinguish pepper accessions from Hunan and Yunnan Guizhou Sichuan regions, and even expand to pepper accession from other areas.

Due to flavonoids being important metabolites that distinguish peppers from different regions, we also focused on the genes related to flavonoid biosynthesis in peppers. Flavonoid 3′-monooxygenase is the core enzyme regulating the aglycone of flavonoid. The gene encoding flavonoid 3′-monooxygenase corresponds to the gene *AT5G07990* in Arabidopsis, and the corresponding homologous genes in peppers are *Cgla01g001199* and *Cgla04g001393* with VIP values greater than 1.5 in the oPLS-DA results, indicating that *Cgla01g001199* and *Cgla04g001393* may regulate flavonoid biosynthesis in peppers, maybe particular related to flavonoids with significant differences in content between the peppers from Hunan and Yun_Gui_Chuan Groups, such as luteolin-6,8-di-C-glucoside, Chrysoeriol 6-C-glucoside (isoscoparin), luteolin-6-C-glucoside (isoorientin), luteolin 6-C-hexoside-8-C-pentoside, luteolin-6-C-2-glucuronylglucoside, luteolin-O-Malonyl-O-hexoside-O-pentoside and apigenin O-hexosyl-O-pentoside.

### 3.3. Characteristic Differential Metabolites and Expressed Genes in Pepepr Accessions from Yunnan, Guizhou and Sichuan Provinces

**Figure 4:**
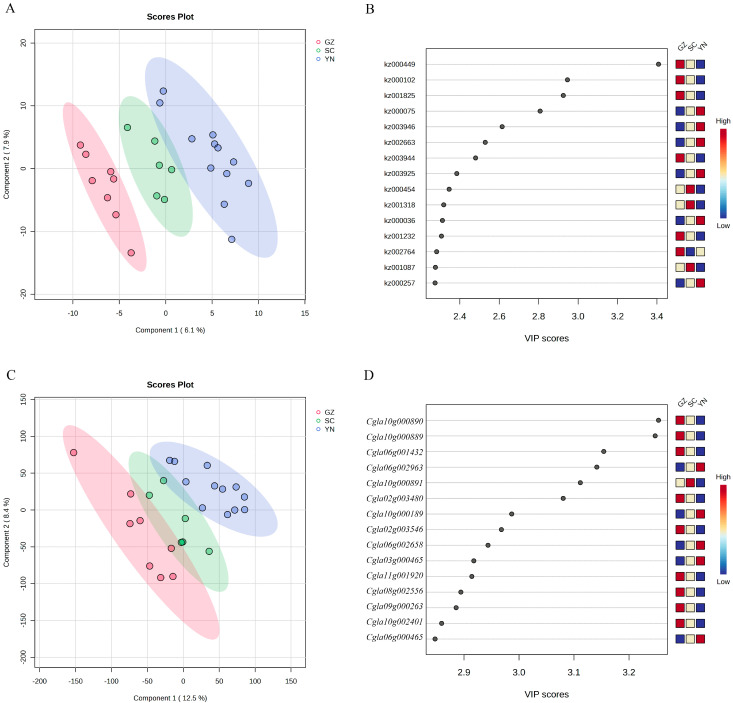
Yunnan, Guizhou and Sichuan pepper accessions transcriptome and metabolome analyses. (**A**): Metabolome PLS-DA score chart; (**B**): Metabolome PLS-DA VIP score chart (**C**): Gene PLS-DA score chart (**D**): Gene PLS-DA VIP score chart.

Although the pepper accessions from Yunnan, Guizhou and Sichuan were very similar in metabolites and transcriptome analysis when compared to pepper accessions from Hunan, we further analyzed the differences in metabolites and transcriptome among pepper accessions from Yunnan, Guizhou and Sichuan. PLS-DA analysis of the metabolites data of pepper accessions from the three provinces showed that pepper accessions from the same province gathered together (Figure 4A), and the accessions from the three provinces can be separated at the horizontal axis. The 15 metabolites with the highest VIP value were four alkaloids, one anthocyanin, four phenolic acids, two organic acids, one saccharide, one terpenoid and two free fatty acids, among which six metabolites (p-Coumaroyl agmatine, Cyanidin 3-Sambubioside, 9-HpOTrE, 13-HOTrE(r), p-coumaroyl malic acid and Nordihydrocapsiate contents are the highest in pepper accessions from Guizhou province, three metabolites (Fer-agmatine, Quinic acid and Maslinic acid) contents are the highest in pepper accessions from Sichuan province, and six metabolites (Caffeic aldehyde, Dihydrocaffeoylglucose, Abscisic acid, N’-p-Coumaroyl agmatine-glucoside, N-(4′-O-glycosyl)-p-coumaroyl agmatine and Ribulose-5-phosphate) contents are the highest in pepper accessions from Yunnan province (Figure 4B; Table 3). Notably, the content of two kinds of free fatty acids, 9-hydroperoxenoic acid (9-HPOTrE) and 13-HOTrE (r), are the highest in pepper accessions from Guizhou province. Free fatty acids play an important role in the flavor formation of many foods, acting directly as the flavor compound and contributing to the formation of the flavor. In addition, the contents of one alkaloid and two phenolic acids are the highest in pepper accessions from Guizhou province. The contents of two alkaloids, two phenolic acids, one organic acid and one saccharide are the highest in pepper accessions from Yunnan province. The contents of one alkaloid, one organic acid and one terpenoid are the highest in pepper accessions from Sichuan province.

The PLS-DA of genes expression data showed that, at the horizontal axis, accessions from the three provinces of Yunnan, Guizhou and Sichuan were separated, and pepper accessions from the same province gathered together (Figure 4C). According to the VIP value, we found that among the 15 genes with the highest VIP value, there were nine genes, *Cgla10g000890*, *Cgla10g000889*, *Cgla06g001432*, *Cgla02g003480*, *Cgla02g003546*, *Cgla11g001920*, *Cgla08g002556*, *Cgla09g000263* and *Cgla10g002401*, expressed highest in pepper accessions from Guizhou province, one gene *Cgla10g000891* expressed highest in pepper accessions from Sichuan province, and five genes, *Cgla06g002963*, *Cgla10g000189*, *Cgla06g000465*, *Cgla06g002658* and *Cgla03g000465* expressed highest in pepper accessions from Yunnan province (Figure 4D, Table 4).

### 3.4. Taste and Nutrition Related Metabolites in Pepper Accession from Four Provinces

**Figure 5:**
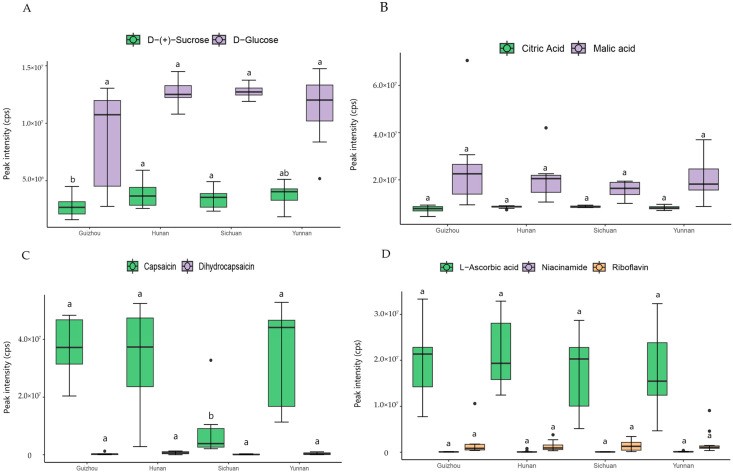
Analyses of taste and nutrition metabolites among Yunnan, Guizhou Sichuan and Hunan province (**A**): sugars; (**B**): acids; (**C**): capsaicinoids; (**D**): vitamins. (Identical lowercase letters indicate no significant difference at the 0.05 level.) Unit: count per second (cps).

In addition to flavonoids, we also conducted comparative analysis on metabolites closely related to the taste and nutrition of pepper, particularly on sugars, acids, capsaicinoids and vitamins. The D- (+) -Sucrose content of accessions from Guizhou province was significantly lower than that of accessions from Hunan and Sichuan, and there was no significant difference from pepper accessions from Yunnan (Figure 5A). The capsaicin of accessions from Sichuan province was significantly lower than that of accessions from the other three provinces, and there was no significant difference in vitamin and acid contents. The citric acid contents of the pepper accessions from the four provinces were similar, while the malic acid content was higher in pepper accessions from Guizhou (Figure 5B). In pepper accessions from Sichuan, the contents of capsaicinoids were lowest. The mean values of capsaicinoids content of pepper accessions from Guizhou, Yunnan and Hunan provinces were similar, but in the pepper accessions from Hunan and Yunnan, the capsaicinoids contents varied considerably; in the pepper accessions from Guizhou, the capsaicinoid contents were similar (Figure 5C). Comparing the mean values of some vitamins’ contents in pepper accessions from different provinces, the average content of riboflavin in accessions from Guizhou was the highest, with high niacinamide and ascorbic acid contents in accessions from Hunan.

## 4. Discussion

Metabolites are important components of plants, such as sugars, proteins, lipids, terpenoids, phenols and alkaloids, which not only play an important role in plant life activities [53], but also are closely related to human life activities [54,55]. As a kind of plants secondary metabolites, flavonoids play important roles in many plants biological processes and in response to environmental factors [56,57]. Environmental factors such as altitude and soil affect changes in plant metabolites [58,59]. For example, the abundant variation in carotenoids was highly associated with high-altitude adaptations in Tibetan peach fruits [60]. In addition, many plants such as the evergreen azalea [61] and prunus fruit tree [62] have adaptive variation in high altitude areas. Yunnan, Guizhou and Sichuan have higher altitudes, which may be responsible for the high content of flavonoids in pepper accessions from Yun_Gui_Chuan Group.

In this study, the differences in the accumulation of fruit metabolites among peppers from the four provinces of China showed that there were significant differences in metabolites between pepper accessions from Yun_Gui_Chuan Group and Hunan Group, particularly in flavonoid accumulation. Pepper accessions from Yun_Gui_Chuan Group had higher contents of nine flavonoids, which were characteristic metabolites through PCA and PLS-DA, and pepper accessions from Hunan Group had only one characteristic flavonoid. The aglycones of the 10 characteristic flavonoids varies in pepper accessions from different groups; in Yun_Gui_Chuan Group, pepper accessions are mainly luteolin and chrysoeriol, and in Hunan Group, the pepper accession is apigenin. In the flavonoid biosynthesis processes, the luteolin and chrysoeriol are located in the downstream of apigenin. It may be that downstream flavonoids (luteolin and chrysoeriol) play a key role in high-altitude adaptation.

After chili peppers were introduced to China, they were commonly grown and eaten throughout the country. The first place where chili peppers began to be consumed was Guizhou, followed by Hunan. People in Guizhou and Hunan almost eat chili peppers for every meal [63], and different tastes are often related to geographical location and customs [64], which is consistent with that the pepper accessions from the two provinces showed relatively high levels of capsaicinoids. In addition, the content of cyanidin 3-Sambubioside, a kind of anthocyanin, is the highest in pepper accessions from Guizhou, which has the inhibitory effect on cancer cell metastasis [65], and quinic acid is the highest in pepper accessions from Guizhou, which have prominent antibacterial activity against bacteria [66].

Previous researchers studied metabolic differences in black pepper among three regions [67], and the results show that fatty acid derivates were the most frequent markers within the metabolites annotated for processing discrimination, such as 10,16-dihydroxyhexadecnoic acid and 9-hydroperoxy-10E-octadecenoic acid. In this study, two kinds of free fatty acids, 9-HPOTrE and 13 (S)-HOTrE, are higher in peppers from Guizhou. Under catalysis by the related enzymes, it can be converted into 9,16-dihydroperoxy acid or colnelenic acid [68]. They may be the important metabolites for the flavor of pepper accessions from Guizhou and can be used as marker metabolites to distinguish peppers from different regions, especially from Guizhou and other regions. Moreover, the previous study was basically about the differences of the same type of pepper in different regions [69,70], while this experiment analyzed the differences among the four provinces as a whole.

In this study, *Cgla06g001871* is related to signal transduction, and a single SNP variant was identified at the position 43,760,475 bp on chromosome 6, which may be developed as a DNA marker to distinguish peppers from different regions, especially from Yun_Gui_Chuan and other regions. Through the detection and analysis of SNP locus information, crops of different varieties or origins can be effectively identified and differentiated, providing strong support for crop genetic improvement and regional specialty variety conservation.

## 5. Conclusions

In this study, according to PCA, PLS-DA, oPLS-DA and FC analyses, we found that pepper accessions from the same province have their own characteristic differential metabolites. In pepper accessions from Hunan Group, the characteristic metabolites are apigenin O-hexosyl-O-pentoside; and in pepper accessions from Yun_Gui_Chuan Group, the characteristic metabolites are also flavonoids. In Yun_Gui_Chuan Group, the characteristic metabolites of pepper accessions from each province were also analyzed. In pepper accessions from Yunnan, the characteristic metabolites are mainly caffeic aldehyde, dihydrocaffeoylglucose, abscisic acid, N’-p-coumaroyl agmatine-glucoside, N-(4′-O-glycosyl)-p-coumaroyl agmatine and ribulose-5-phosphate. In pepper accessions from Guizhou, the characteristic metabolites are mainly p-coumaroyl agmatine, cyanidin 3-sambubioside, 9-HpOTrE, 13-HOTrE(r), p-coumaroyl malic acid and nordihydrocapsiate. In pepper accessions from Sichuan, the characteristic metabolites are mainly fer-agmatine quinic acid and saslinic acid.

We found the characteristic expressed genes in the four provinces. The expression of these eight genes *Cgla04g002509*, *Cgla05g001108*, *Cgla06g001871*, *Cgla06g002771*, *Cgla06g002772*, *Cgla07g000433*, *Cgla07g000244* and *Cgla03g0033162*, were higher in accessions from Hunan Group, and *Cgla08g002247*, *Cgla08g002205*, *Cgla02g000694*, *Cgla04g000060*, *Cgla01g002925*, *Cgla05g000982* and *Cgla12g001321* were higher in the pepper accessions from Yun_Gui_Chuan Group. Notably, an SNP was found in gene *Cgla06g001871* with strong correlation with pepper accessions from Yun_Gui_Chuan Group, which can be served as a marker for identifying the source of pepper accessions. In Yun_Gui_Chuan Group, the characteristic expressed genes of pepper accessions from each province were also analyzed. There are nine genes, *Cgla10g000890*, *Cgla10g000889, Cgla06g001432*, *Cgla02g003480*, *Cgla02g003546*, *Cgla11g001920*, *Cgla08g002556*, *Cgla09g000263* and *Cgla10g002401*, expressing highest in accessions from Guizhou province; one gene, *Cgla10g000189*, expressing highest in accessions from Sichuan province and five genes, *Cgla06g002963*, *Cgla10g000189*, *Cgla06g000465*, *Cgla06g002658* and *Cgla03g000465*, expressing highest in accessions from Yunnan. The flavonoid 3′-monooxygenase corresponds to the gene *AT5G07990* in Arabidopsis, and the corresponding homologous genes in peppers are *Cgla01g001199* and *Cgla04g001393* with VIP values greater than 1.5 in the oPLS-DA results, indicating that *Cgla01g001199* and *Cgla04g001393* may be regulating flavonoid biosynthesis in peppers. This study lays the foundation for characteristic metabolites and expressed genes in pepper accessions from different regions.

## Figures and Tables

**Table 1:** Differential metabolites of Hunan and Yun_Gui_Chuan Groups, analyzed by all three methods (PCA, oPLSDA and FC analyses).

KZ ID	Compounds	Class	The Group from Which Pepper Accessions with Higher Characteristic Metabolites Content Come
kz002597	Kaempferol-C-glucoside-C-glucoside	Flavonoid carbonoside	Yun_Gui_Chuan
kz000935	C-Hexosyl-luteolin C-pentoside	Flavonoid carbonoside	
kz000832	Luteolin-6,8-di-C-glucoside	Flavonoid carbonoside	
kz002593	luteolin-6-C-glucoside (Isoorientin)	Flavones	
km1214	Chrysoeriol 8-C-hexoside	Flavonoid carbonoside	
kz000927	Luteolin-6-C-glucoside (Isoorientin)	Flavonoid carbonoside	
Rfa072N	Luteolin 6-C-hexoside-8-C-pentoside	Flavonoid carbonoside	
kz000835	Luteolin-6-C-2-glucuronylglucoside	Flavonoid carbonoside	
kz003987	Luteolin-O-Malonyl-O-Hexoside-O-Pentoside	Flavones	
kz000820	Apigenin O-hexosyl-O-pentoside	Flavones	Hunnan

**Table 2:** Functional annotation of the differential expressed genes between Hunan Group and Yun_Gui_Chuan Group with the top 15 VIP value.

Gene ID	Function Annotation	The Group from Which Pepper Accessions with Higher Characteristic Gene Expression Come
*Cgla01g002925*	F-box domain-containing protein	Yun_Gui_Chuan
*Cgla02g000694*	Transcription factor TCP17	
*Cgla04g000060*	RING-H2 finger protein ATL44	
*Cgla05g000982*	sulfated surface glycoprotein 185-like	
*Cgla08g002205*	F-box domain-containing protein	
*Cgla08g002247*	F-box domain-containing protein	
*Cgla12g001321*	Rop guanine nucleotide exchange factor 9	
*Cgla03g003316*	Transposon MuDR mudrA-like protein, putative	Hunan
*Cgla04g002509*	Chaperone protein dnaJ 2	
*Cgla05g001108*	protein PHYLLO, chloroplastic isoform X1	
*Cgla06g001871*	Phorbol-ester/DAG-type domain-containing protein	
*Cgla06g002771*	Putative α-mannosidase I MNS5	
*Cgla06g002772*	Unknown function	
*Cgla07g000244*	Pentatricopeptide repeat-containing protein, chloroplastic	
*Cgla07g000433*	START domain-containing protein	

**Table 3:** Characteristic differential metabolites of accessions from Yunnan, Guizhou and Sichuan provinces.

Kz ID	Compounds	Class	The Province from Which Pepper Accessions with the Highest Characteristic Metabolites Come
kz000449	p-Coumaroyl agmatine	Alkaloids	Guizhou
kz002764	Cyanidin 3-Sambubioside	Anthocyanins	
kz000102	9-HpOTrE	Free fatty acids	
kz001232	13-HOTrE(r)	Free fatty acids	
kz001825	p-coumaroyl malic acid	Phenolic acids	
kz003944	Nordihydrocapsiate	Phenolic acids	
kz000454	Fer-agmatine	Alkaloids	Sichuan
kz001318	Quinic acid	Organic acids	
kz001087	Maslinic acid	Terpenoids	
kz000075	Caffeic aldehyde	Phenolic acids	Yunnan
kz003946	Dihydrocaffeoylglucose	Phenolic acids	
kz002663	Abscisic acid	Organic acids	
kz003925	N’-p-Coumaroyl agmatine-glucoside	Alkaloids	
kz000036	N-(4′-O-glycosyl)-p-coumaroyl agmatine	Alkaloids	
kz000257	Ribulose-5-phosphate	Saccharides	

**Table 4:** Differential expressed genes of accessions from Yunnan, Guizhou and Sichuan provinces.

Gene	Function Annotation	The Province from Which Pepper Accessions with the Highest Characteristic Metabolites Come
*Cgla10g000889*	3-deoxy-manno-octulosonate cytidylyltransferase, mitochondrial	Guizhou
*Cgla02g003480*	Protein kinase domain-containing protein	
*Cgla02g003546*	Putative pentatricopeptide repeat-containing protein *At3g01580*	
*Cgla06g001432*	gibberellin 3-β-dioxygenase 1-like	
*Cgla08g002556*	Uncharacterized protein	
*Cgla09g000263*	Uncharacterized protein	
*Cgla10g000890*	DUF547 domain-containing protein	
*Cgla10g002401*	GH10 domain-containing protein	
*Cgla11g001920*	protein phytochrome kinase substrate 1	
*Cgla10g000891*	DUF547 domain-containing protein	Sichuan
*Cgla03g000465*	Peptidase_M16_C domain-containing protein	Yunnan
*Cgla06g000465*	CRAL-TRIO domain-containing protein	
*Cgla06g002658*	Dehydroascorbate reductase	
*Cgla06g002963*	Serine/threonine-protein kinase HT1-like (Fragment)	
*Cgla10g000189*	Unknown function	

## Data Availability

The original contributions presented in the study are included in the Supplementary Tables S5 and S6.

## Supplementary Materials

The following supporting information can be downloaded at: https://www.mdpi.com/article/10.3390/genes16020137/s1, Table S1: Summary of the sampled collection of pepper.; Table S2: The 584 metabolites coefficients of variation (CV) less than 30% in the quality control.; Table S3: Top 15 metabolites with the highest VIP values.; Table S4: The differential metabolites of pepper accessions from Hunan and Yun_Gui_Chuan Group.

## Abbreviations

The following abbreviations are used in this manuscript:

FAO	Food and Agriculture Organization of the United Nations
PCA	Principal Component Analysis
FC	Fold Change
FDR	False Discovery Rate
VIP	Variable Importance in the Projection
oPLS-DA	orthogonal Partial Least Squares Discriminant Analysis
PLS-DA	Partial Least Squares Discriminant Analysis
CV	Coefficients of Variation
QC	Quality Control
